# Population Density and Distribution of Wheat Bugs Infesting Durum Wheat in Sardinia, Italy

**DOI:** 10.1673/031.013.5001

**Published:** 2013-06-06

**Authors:** Luigi Salis, Marta Goula, Jordi Izquierdo, Elena Gordún

**Affiliations:** 1 Departament de Biologia Animal, Facultat de Biologia, Universitat de Barcelona, Spain.; 2 IRBiO Institut de Recerca de la Biodiversitat, Universitat de Barcelona, Spain; 3 Departament d'Enginyeria Agroalimentària i Biotecnologia, Escola Superior d'Agricultura de Barcelona, Universität Politècnica de Catalunya, Spain

**Keywords:** *Aelia*, density, *Eurygaster*, Hemiptera-Heteroptera, sunn pest

## Abstract

Wheat is a very important crop in Italy, and is infested by wheat bugs belonging to the genera *Eurygaster* (Hemiptera: Scutellaridae) and *Aelia* (Hemiptera: Pentatomidae). Many wheat bug infestations have been reported in the north, south, and center of Italy, both in the past as well as recently. The present study was carried out in Sardinia, Italy, during two years (2007 and 2008). The objective of this study was to determine the species and distribution of wheat bugs in durum wheat fields in Sardinia, and to estimate their population density in order to know the incidence of the pest on the island. Sampling took place twice a year (May and June) in three zones, representative of durum wheat cropping in the island. Four species of wheat bugs were found; the predominant species was *Eurygaster austriaca* (Schrank), followed by *Aelia germari* (Kuster), *Eurygaster maura* L., and *Aelia acuminata* L. The average density of wheat bugs was low (1.1 individuals/m^2^), but in certain areas it was above the damage threshold (4 individuals/m^2^). For this reason, the conclusion of the study is that this pest should be monitored in order to control outbreaks and prevent their further spread.

## Sommario

Il frumento è una coltura molto importante in Italia e non è esente da infestazioni di cimici dei cereali appartenenti ai generi *Eurygaster* (Hemiptera: Scutellaridae) e *Aelia* (Hemiptera: Pentatomidae). Molte infestazioni di cimici dei cereali sono state segnalate nel Nord, Sud e Centro Italia, nel passato come di récente. Questo studio è stato condotto in Sardegna (Italia) nel corso di due anni (2007 e 2008). L'obiettivo di questo studio è determinare le specie e la distribuzione délle cimici dei cereali presenti nei campi di frumento duro in Sardegna, e stimare la loro densità di popolazione al fine di conoscere l'incidenza di questi insetti nocivi nell'isola. Il campionamento ha avuto luogo due volte l'anno (maggio e giugno) in tre zone, rappresentative della coltura del frumento duro dell'isola. Quattro specie di cimici dei cereali sono state trovate, la specie predominante era *l'Eurygaster austriaca* (Schrank), seguita da *Aelia germari* (Kuster), *Eurygaster maura* L. e *Aelia acuminata* L. La densità media delle cimici dei cereali è stata bassa (1,1 individui/m^2^) , ma in alcune zone è stata al di sopra della soglia di danno (4 individui/m^2^). Per questo motivo, la conclusione dello studio èche le cimici dei cereali devono essere monitorate per controllare e prevenire i focolai e la loro ulteriore diffusione.

## Introduction

The names “sunn pest” and “wheat bug” refer to different species in the genera *Eurygaster* (Hemiptera: Scutellaridae) and *Aelia* (Hemiptera: Pentatomidae). Wheat bugs are widely distributed in various areas of Europe, Asia, and North Africa (Paulian and Popov 1980). An estimate of more than 15 million hectares of cereal, mainly wheat and barley, are infested annually in Syria, Iraq, Iran, Turkey, Afghanistan, and Lebanon, as well as in Central Asia and the Caucasus, Bulgaria, and Romania (El Bouhssini et al. 2002).

The genus *Eurygaster* includes 15 species (Froeschner 1988a; Javahery et al. 2000; Göllner-Scheiding 2006), of which only three are cereal pests, namely *Eurygaster integriceps* (Puton), *Eurygaster maura* L., and *Eurygaster austriaca* (Schrank) (Paulian and Popov 1980). *E. integriceps* is found from southern Europe up to China, although it is absent from the Iberian Peninsula; *E. maura* is present in Europe, North Africa, and central Asia; and finally, *E. austriaca* extends across the Euromediterranean region up to central Asia (Göllner-Scheiding 2006).

The genus *Aelia* includes 16 species (Froeschner 1988b; Derjanschi and Péricart 2005; Rider 2006), of which both *Aelia acuminata* L. and *Aelia rostrata* (Boheman) are known to be important cereal pests. In addition, *Aelia germari* (Kuster) and *Aelia klugii* (Hahn), among other *Aelia* species, can cause occasional damage (Paulian and Popov 1980). Both *A. acuminata* and *A. klugii* are Palaearctic species, and *A. rostrata* is present in the Euromediterranean region, extending eastwards up to India, whereas *A. germari* is found only in the Mediterranean basin (Rider 2006).

Wheat bug populations are generally univoltine, with the exception of certain *Aelia* species (Javahery 1996). In the spring, adults that have overwintered copulate and oviposit in the cereal fields, and the new generation of adult wheat bugs appears after going through five nymphal stages (Voegelé 1996). In autumn and winter, these adults undergo diapause after migrating over considerable distances or dispersing locally to overwintering sites (Brown 1965; Javahery 1996; Voegelé 1996). *Aelia* spp. and *Eurygaster* spp. undergo obligate diapause throughout their geographical range, and the diapause is influenced both by photoperiod and temperature (Javahery 1996). They hibernate as adults in various shelters including stones, dry leaves, and grass clumps (Voegelé 1996). All wheat bugs overwinter until temperatures rise in spring, at which time they move to cereal fields to feed and mate. The adults die soon after completing oviposition. Feeding in spring is essential prior to the first mating and oviposition for both sexes (Javahery 1996). Some *Eurygaster* and *Aelia* species are strongly migratory (> 20 km) while others are sedentary or only subject to very minor dispersion. Whether or not *Eurygaster* spp. and *Aelia* spp. invade areas that appear to be ecologically suitable may be explained according to wind direction (Brown 1965). Damage to the crop is proportional to the density of wheat bugs. Population density is directly related to hibernation success, which in turn depends on the accumulation of fat reserves prior to hibernation (Donkstoff 1996). Changes in population densities and outbreaks of these insects are largely determined by external abiotic and biotic factors. Climatic conditions, especially temperature and rainfall, play an important role in the population dynamics of wheat bugs. Continuous rainfall delays wheat bug activity, and long periods of high humidity in the overwintering sites cause mortality (Javahery 1996). Among the natural enemies observed, the most important belong to Hymenoptera, Diptera, and Fungi (Voegelé 1996) ,and they contribute to the regulation of wheat bug populations. Field margins are the main source of many natural enemies of this pest (Tshernyshev et al. 2010).

The economic importance of wheat bug damage is due to crop losses and/or quality loss of wheat (Kinaci and Kinaci 2004), semolina (Ozderen et al. 2008; Köksel et al. 2009; Salis et al. 2010), or flour (Hariri et al. 2000; Sivri et al. 1999, 2004; Aja et al. 2004; Vaccino et al. 2006; Werteker and Kramreither 2008). The feeding activity of wheat bugs also heavily affects the germination percentage of wheat (Bin et al. 2006). Both nymphs and adults of *Eurygaster* spp. and *Aelia* spp. cause a reduction of wheat quality when they insert their piercing-sucking mouthparts in the kernels and extract the substances within. In order to facilitate the suction of the nutritional elements of the endosperm, the kernels are digested externally by injecting saliva rich in proteases (Sivri et al. 1998; Konarev et al. 2011) and amylases (Kazzazi et al. 2005). The detrimental effect of such proteases on baking quality is very high, even when only 3–5% of kernels are damaged, and dramatically increases for values higher than 10% (Karababa and Ozan 1998; Hariri et al. 2000).

In Italy, wheat is not free of wheat bugs. Malenotti (1931) reported a heavy infestation of *A. acuminata* in 1931 in the province of Verona, and *E. maura* and *Eurygaster hottentota* F. were also found. In 1932–1933, a heavy infestation of *A. rostrata* was recorded in the provinces of Verona, Mantova, and Brescia, and *E. maura* was also found (Malenotti 1933). Less important infestations have been registered in the south of Italy, particularly in the Puglia region (Genduso and Di Martino 1974). Severe infestations of *A. rostrata*, together with the presence of *E. maura* and *E. austriaca*, were registered in Sicily in 1973–1975 (Genduso 1977). In 1998–1999, significant attacks of *E. maura*, and to a lesser extent *E. austriaca* and *Aelia* spp., were reported in Piedmont and on localities in the provinces of Alessandria and Asti (Tavella and Migliardi 2000). In 2000, *A. rostrata* was recorded in Sicily (Spina 2000). In 2005, a heavy infestation of *Eurygaster* spp. on soft wheat occurred in central Italy (Val di Chiana, Toscana) and required an insecticide treatment (Bin et al. 2006).

Durum wheat (*Triticum turgidum* L. var *durum*) is one of the most important crops in Italy, a country that generates around 50% of the total durum wheat production of the European Union, and around 15% of the world production (Sgroi and Fazio 2008). In 2008, Italy produced approximately 5.2 million tonnes of durum wheat (ISTAT 2010). Durum wheat constitutes ∼70% of the total area cultivated with wheat in Italy. In Sardinia, durum wheat is the most widespread crop; it is grown on about 84,000 hectares, with an average production of about 134,000 tonnes per year (ISTAT 2010). Durum wheat is a very ancient crop in the Mediterranean basin and is used mainly to manufacture pasta, as well as for baking traditional types of bread (Quaglia 1988) with a particular interest both from an economic and a cultural point of view (Dexter and Marchylo 2000); the carasau bread, for example, made from durum wheat, is one of the most important products of the Sardinian bread making tradition (Dettori et al. 2002).

No studies on the distribution and density of wheat bugs have been carried out in Sardinia. Considering the importance of durum wheat in the Sardinian economy, a detailed knowledge of the species’ distribution is required as a first step to develop sustainable management options for the improvement of the quality of wheat.

**Figure 1.**
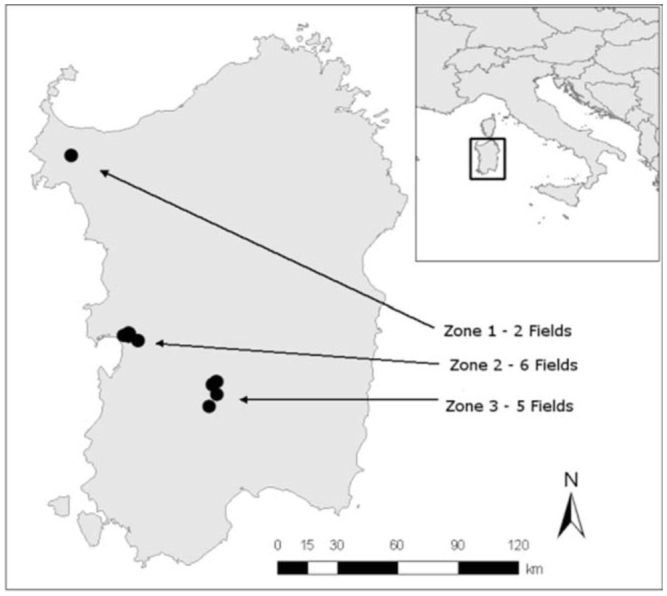
Zones and location of the fields sampled in Sardinia, Italy. High quality figures are available online.

The aim of this study was to determine the species of wheat bugs present in the durum wheat fields of Sardinia, Italy, to explore their distribution in the island, and to estimate their population density in order to know the incidence of the pest on the island.

**Table 1.**
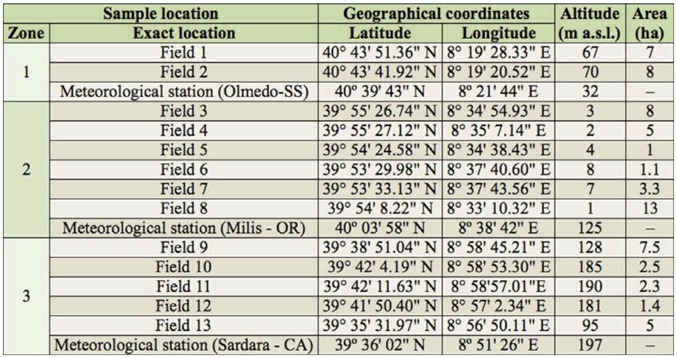
Geographic coordinates, altitude, and areas of sampled fields and meteorological stations of each zone.

## Material and Methods

The survey of wheat bugs was conducted during 2007 and 2008 in 13 durum wheat fields distributed in three different zones (Zone 1, Zone 2, and Zone 3), which were representative of durum wheat cropping in Sardinia (Figure 1; Table 1). Fields were selected at random within each zone. The number of sampled fields in each zone was proportional to the cultivated area. In the surveyed fields, neither pesticides nor fungicides were used, according to common agricultural practice in the region. In each field, insects were collected along six transects. Three transects covered the entire field edge, and the other three covered the interior of the field, following the protocol described by Pérez-Rodríguez et al. (2008). The field edge was considered to be the area between the border of field and two linear meters into the field, while the remaining part of the field was considered to be the interior. Along each transect, insects were collected in 15 regularly spaced sampling points. At each sampling point, an entomological sweep net with an opening of 0.17 m^2^ was swept once over the cereal spikes in order to collect bugs. In other words, the total area sampled per transect was 2.55 m^2^. Considering six transects per field, a total of 90 points were sampled, equivalent to 15.3 m^2^ sampled per field. Sampling took place twice a year, at the beginning of grain filling (1–10 May) to account for the initial population of bugs, and at grain maturation stage (10–20 June), just before harvest, to account for the bugs’ final population. The insects collected were kept separately according to transect. Insects were preserved with ethyl acetate to keep them in good condition until identification of species and development stage (adults and nymphs) in the laboratory. The species were identified under the binocular microscope, and genitalia were studied when necessary. Species were identified according to Vidal (1949), Stichel (1957), Kis (1984), Tamanini (1988), and Ruiz et al. (2003).

**Figure 2.**
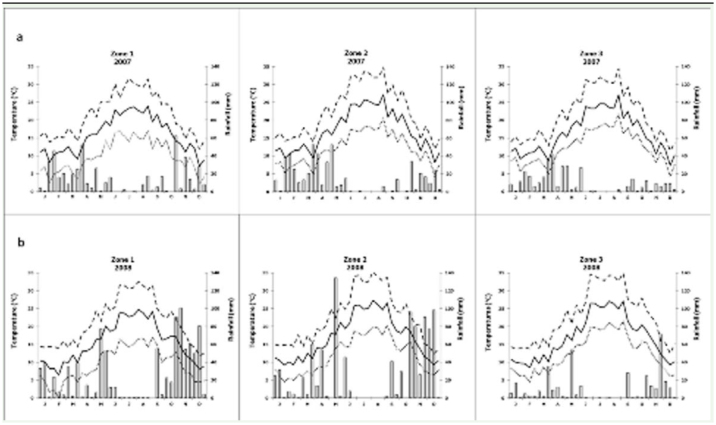
Rainfall (bars), average (solid line), maximum (dashed line), and minimum (dotted line) temperatures from 1 January to 31 December in 2007 (a) and 2008 (b). Rainfall values are sums, and temperature values are means, over 10-day periods. High quality figures are available online.

Meteorological data of each zone were taken from the nearest meteorological stations to the sampled fields (Table 1). Maximum and minimum temperature and rainfall were recorded daily. The study area is characterized by a typical Mediterranean climate with long, hot, dry summers and short, mild, rainy winters. The climatic variables measured for the three zones in 2007 and 2008 are reported in Figure 2. The rainfall for Zone 1 and Zone 2 was very similar within the same year, around 500 mm in 2007 and around 900 mm in 2008. Zone 3 was much drier both years, with a rainfall of 350 mm. In 2007, Zone 2 registered the highest mean temperature (17.1° C) with respect to Zone 3 (16.3° C) and Zone 1 (15.8° C). In 2008, Zone 2 and Zone 3 registered the same mean temperature (16.9° C), while Zone 1 was the coldest zone both years with a mean temperature of 15.5° C.

Differences (in individuals/m^2^) between species, zones, sampling dates, development stage, and field zone were determined by fitting a generalized linear model to the data and estimating the dispersion parameter by maximum likelihood using the procedure GENMOD from the SAS software package (SAS Institute 2009) with log as a link function. Species, zone, sampling date, and field zone were considered to be fixed factors. Differences between means were computed, analyzing multiple pairwise differences with Tukey's test. Analyses for differences in insect densities between field zones (interior and edge) based on transect data showed no significance, and consequently, density data from all transects were pooled by field. Subsequent statistical analyses were performed using insect densities per field.

**Table 2.**
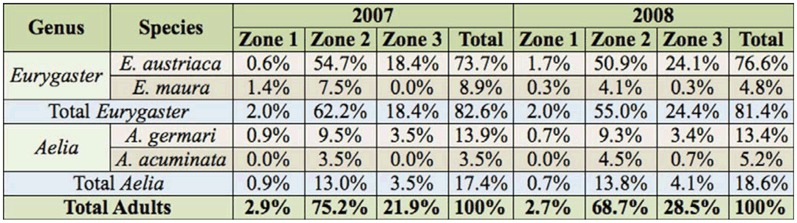
Percentage of the different species of the genus *Eurygaster* and *Aelia* collected per year and zone. Values are expressed in percentage of the total number of adults captured during each year (n = 347 in 2007; n = 291 in 2008).

## Results and Discussion

### Genera and species of wheat bugs in Sardinia, and geographic distribution

During the two years of sampling in the 13 durum wheat fields of Sardinia, two genera, *Eurygaster* and *Aelia*, and four species, *E. austriaca, E. maura, A. germari*, and *A. acuminata*, were identified. Other species of *Eurygaster* and *Aelia* that have been cited in Sardinia, such as *E. hottentotta, A. rostrata, Aelia notata* (King), and *A. klugii*, were not found. Other species reported in other parts of Italy (Servadei 1952; Tamanini 1988; Faraci and Rizzotti Vlach 1995; Derjanschi and Péricart 2005; Fauna Europaea 2011), such as *E. integriceps, Eurygaster testudinaria* (Geoffroy), *Eurygaster dilaticollis* (Dohrn), and *Aelia sibirica* (Reuter), were not found either.

**Figure 3.**
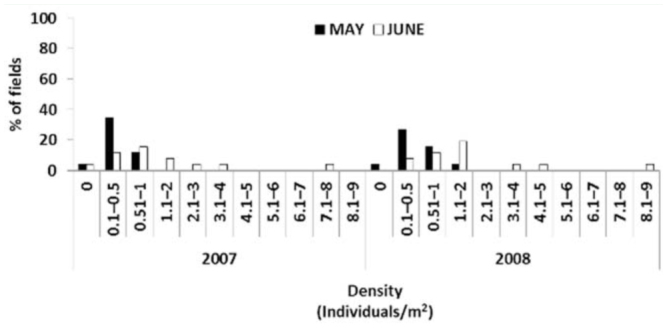
Frequency classes of wheat bug densities (individuals/m^2^) found in fields sampled in May and June in 2007 and 2008. High quality figures are available online.

The results regarding the different species collected from each zone are shown in Table 2, in which data is expressed as percentage of total adults captured per year. The most abundant species was *E. austriaca*, which represented 75.1% of the total number of adults collected, followed by *A. germari* (13.6%), *E. maura* (7.1%), and *A. acuminata* (4.2%). These four species were present in all zones except for *A. acuminata*, which was never found in Zone 1. In all the zones and years, the most abundant species was *E. austriaca*, with an exception in Zone 1 in 2007, where *E. maura* was the most abundant species.

### Density of wheat bugs in durum wheat fields

#### Frequency classes of wheat bug densities

In 2007 and 2008, about 80% and 65% respectively of the fields sampled registered a very low density of wheat bugs, between 0 and 1 individuals/m^2^ (Figure 3). In June in both years, the density of wheat bugs was higher compared to May, and approximately half of the fields registered a density that ranged from 0.5 to 2 individuals/m^2^ (Figure 3). There were very few fields with densities above 4 individuals/m^2^, established as the damage threshold by Paulian and Popov (1980). The exceptions were observed in Zone 2, which had 4.3 and 7.3 individuals/m^2^ in field 3 in June 2007 and 2008 respectively, and 8.2 individuals/m^2^ in field 8 in June 2008.

**Figure 4.**
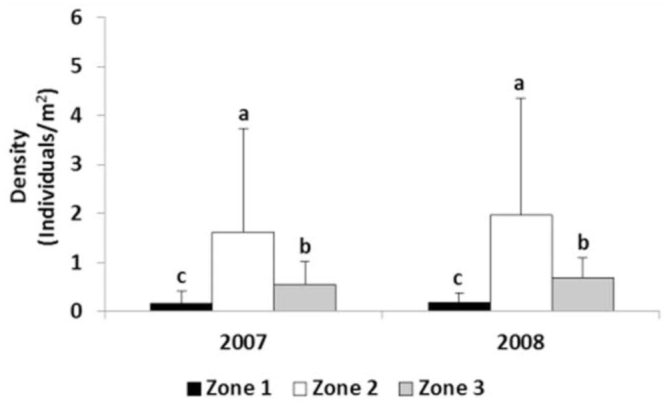
Mean densities of wheat bugs per year in the different zones. In each year, the same letter indicates no significant differences (*p* < 0.05). Bars represent standard deviation (n = 312). High quality figures are available online.

#### Total density of wheat bugs per year and zone

A total of 867 wheat bugs (638 adults and 229 nymphs) were collected. The average density of wheat bugs sampled in Sardinia in 2007 (0.98 individuals/m^2^) was not significantly different (*p* > 0.05) than 2008 (1.19 individuals/m^2^). Population densities, however, varied significantly between zones (Figure 4). The average density in Zone 2 was significantly higher than Zone 3 and Zone 1 (Figure 4). Many factors influence the optimal conditions for the development of wheat bugs, such as climatic conditions, areas cropped, parasites, and overwintering sites (Javahery 1996; Popov et al. 2003; Kutuk et al. 2010), and they can determine different population densities. During the period 20 April - 20 June, immediately before the first sampling (1–10 May) until the second sampling (10–20 June), the average temperature in Zone 2 was the highest (in 2007: Zone 2, 19.3° C; Zone 3, 18.7° C; Zone 1, 18.5° C; in 2008: Zone 2, 18.7° C; Zone 3, 18° C; Zone 1, 17.5° C). In both years, during the period considered above, the average maximum temperature was highest in Zone 2, whereas Zone 1 always presented the lowest average minimum temperature. Higher temperatures stimulate the development of wheat bugs (Javahery 1996; Iranipour et al. 2010) and could explain the different densities in the three zones (Figure 4).

**Figure 5.**
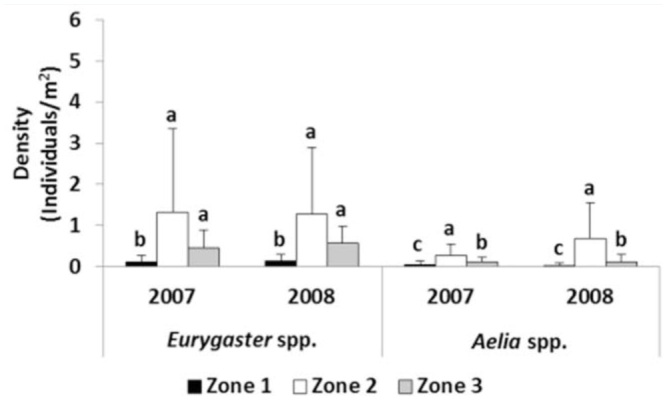
Mean densities of *Eurygaster* spp. and *Aelia* spp. per year and zone (B). Within each genus and year, the same letter indicates no significant differences (p < 0.05). Bars represent standard deviation (n = 31 2). High quality figures are available online.

The most abundant genus, statistically significant in both years of the study, was *Eurygaster*, with 0.80 individuals/m^2^ in 2007 and 0.83 individuals/m^2^ in 2008, while *Aelia* had a density of 0.18 individuals/m^2^ and 0.37 individuals/m^2^ in 2007 and 2008 respectively (*p*<0.05).

**Figure 6.**
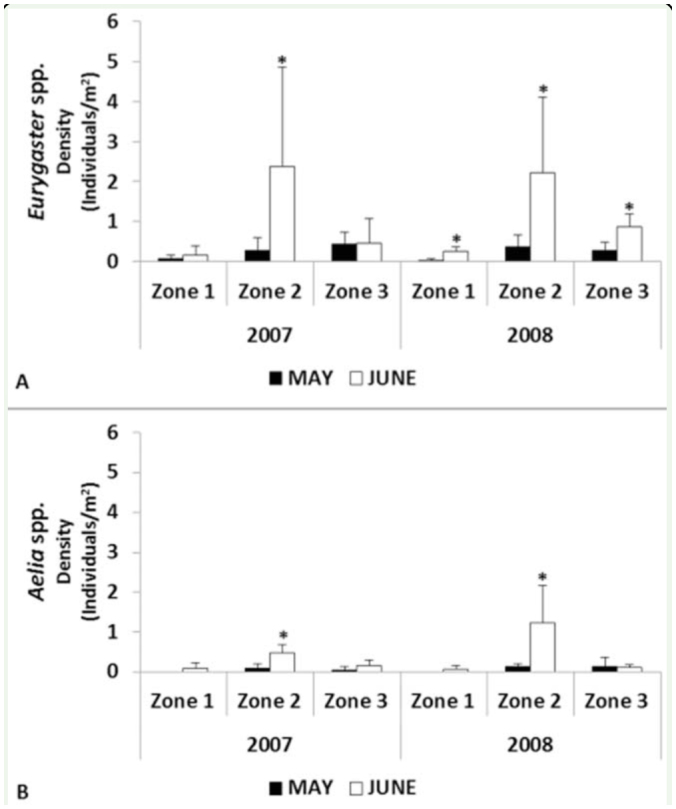
Mean density of *Eurygaster* spp. (A) and *Aelia* spp. (B) per year in the different zones in May and June. Data followed by an asterisk (*) indicates significant differences (*p* < 0.05) within each zone in each year. Bars represent standard deviation (n = 312 for each genus). High quality figures are available online.

The population density of each genus (Figure 5) showed similar results as total density. In both years, the density of the genus *Eurygaster* was significantly higher in Zone 2 and Zone 3 compared to Zone 1. Similarly, the density of the genus *Aelia* was always significantly higher in Zone 2 than in Zone 3 and Zone 1. The density of *Aelia* was higher in Zone 3 than in Zone 1 in 2007 and 2008 (Figure 5).

#### Density of wheat bugs by sampling date (May-June) and by development stage (nymphs and adults)

Densities of wheat bugs were statistically lower (*p* < 0.05) in the first sampling date (May) than in the second sampling date (June) in Zone 1 (in 2007, 0.07 individuals/m^2^ and 0.26 individuals/m^2^ respectively; in 2008, 0.03 individuals/m^2^ and 0.33 individuals/m^2^ respectively) and Zone 2 (in 2007, 0.38 individuals/m^2^ and 2.84 individuals/m^2^ respectively; in 2008, 0.49 individuals/m^2^ and 3.45 individuals/m^2^ respectively). According to the wheat bugs’ life-cycle, they overwinter in or under diverse shelters (stones, dry leaves, grass clumps) until they move to cereal fields to feed and mate. Few adults were found in wheat fields in May (< 1 individuals/m^2^). No nymphs were found in May, as reproductive activity had not yet begun. It is important to know the density of the overwintered adults in wheat fields because it is associated to nymphs and new-adults generation (Kutuk et al. 2010), which is the most detrimental to wheat crop.

**Figure 7.**
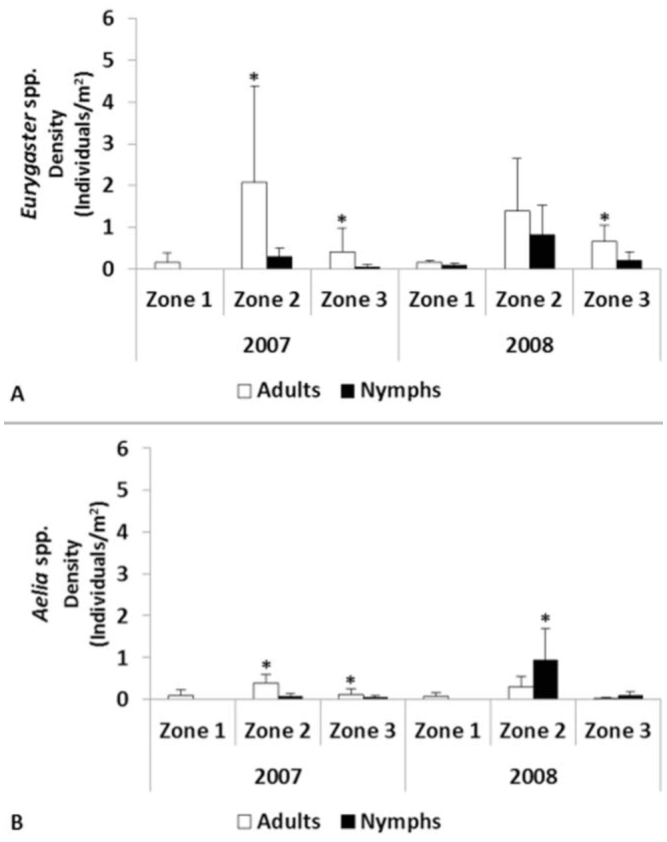
Mean densities of adults and nymphs of *Eurygaster* spp. (A) and *Aelia* spp. (B) in June. Data followed by an asterisk (^*^) indicates significant differences (*p* < 0.05) within each zone in each year. Bars represent standard deviation (n = 3 12 for each genus). High quality figures are available online.

In June, when wheat was at the end of grainfilling and maturation, overwintered adults had already reproduced and a mixed population of nymphs and adults was found, resulting in a higher population density with respect to May, as explained above. These findings, regarding wheat bugs densities in May and June, agree with other studies, such as Hariri et al. (2000) in Syria, Popov et al. (2003) in Romania, and Kutuk et al. (2010) and Canhilal et al. (2005) in Turkey.

The genus *Eurygaster* showed significantly higher densities (*p* < 0.05) in June than in May only in Zone 2 in 2007 and in all the zones in 2008 (Figure 6A). The densities of the genus *Aelia* were significantly higher in Zone 2 in June of both years (Figure 6B).

As regards the density of adults and nymphs in June, significantly more *Eurygaster* spp. adults than nymphs were recorded in Zone 2 in 2007 and in Zone 3 both years (Figure 7A). For *Aelia* spp., significantly more adults than nymphs were found in Zone 2 and Zone 3 in 2007 (Figure 7-B). The only exception is for*Aelia* spp. in Zone 2 in 2008, when significantly more nymphs than adults were found (Figure 7B). Finally, in some cases no nymphs were recorded from the genus *Eurygaster* in Zone 1 in 2007 and from the genus *Aelia* in Zone 1 for both years (Figure 7AB).

#### Density by field zone: edge or interior

No significant differences in density between the edge and the interior of the field were found for either *Eurygaster* spp. or *Aelia* spp., neither in any of the years studied nor in the two sampling periods, except *for Aelia* spp. in June 2007 in Zone 2, in which its density was significantly higher (*p* < 0.05) in the edge of the field (0.63 individuals/m^2^) than in the interior (0.31 individuals/m^2^). This result was possibly due to the fact that many sampled fields were side by side in a continuum without a properly limiting edge. Conversely, in other studies (Afonina et al. 2001; Pérez-Rodriguez et al. 2008) in which the wheat fields were sampled in the edge and in the interior, differences were found between the field zones. In a region of South Russia, for example, *E. integriceps* began to colonize the field from its edges; however, the following generations were more abundant in the center of the field (Afonina et al. 2001). In northeast Spain, differences between density in the edge and the interior of the field were found only for *Aelia* spp., but not for *Eurygaster* spp. (Pérez-Rodriguez et al. 2008). It is important to know the distribution of the wheat bugs in fields in order to select the appropriate sampling method to be used. This could, moreover, permit the early detection of the wheat bugs before copulation and oviposition, which is very important for the control of this pest.

### Conclusions

The predominant species of wheat bug found in the durum wheat fields sampled in Sardinia was *E. austriaca*, followed by *A. germari, E. maura*, and *A. acuminata*. Bug density varied significantly according to the zone, being much higher in Zone 2 than in Zone 1 and Zone 3. The average density of bugs was low (1.1 individuals/m^2^), but in certain areas it was above the damage threshold (4 individuals/m^2^). Therefore, it would be necessary to monitor the wheat bugs in order to detect outbreaks before they produce economic damage and spread to other areas. The overall density of wheat bugs was lower in May than in June. No nymphs were found in May, only adults. No significant differences were found in the distribution of bugs between field edge and interior, except for *Aelia* spp. in Zone 2 in 2007.

Considering how important durum wheat crops are to the Italian economy, and in view of the infestations of wheat bugs in several Italian regions, it would be desirable to carry out more studies on wheat bugs in the durum wheat production areas. A better understanding of the spatial-temporal population trends is needed in order to develop and apply a costeffective and environmentally sound pest management program for the control of these pests.
